# BDPapayaLeaf: A dataset of papaya leaf for disease detection, classification, and analysis

**DOI:** 10.1016/j.dib.2024.110910

**Published:** 2024-09-10

**Authors:** Sumaya Mustofa, Md Taimur Ahad, Yousuf Rayhan Emon, Arpita Sarker

**Affiliations:** Daffodil International University, Savar, Dhaka 1340, Bangladesh

**Keywords:** Dataset, Directory, Anthracnose, Curl, Spot, Annotation, Label, Plant

## Abstract

Papaya is a popular vegetable and fruit in both developing and developed countries. Nonetheless, Bangladeshʼs agricultural landscape is significantly influenced by papaya cultivation. However, disease is a common impediment to papaya productivity, adversely affecting papaya quality and yield and leading to substantial economic losses for farmers. Research suggests that computer-aided disease diagnosis and machine learning (ML) models can improve papaya production by detecting and classifying diseases. In this line, a dataset of papaya is required to diagnose the disease. Moreover, like many other fruits, papaya disease may vary from country to country. Therefore, the country-based papaya disease dataset is required. In this study, a papaya dataset is collected from Dhaka, Bangladesh. This dataset contains 2159 original images from five classes, including the healthy control class and four papaya leaf diseases: Anthracnose, Bacterial Spot, Curl, and Ring spot. Besides the original images, the dataset contains 210 annotated data for each of the five classes. The dataset contains two types of data: the *whole image* and the *annotated image*. The image will interest data scientists who apply disease detection through a convolutional neural network (CNN) and its variants. Furthermore, the annotated images, such as You Only Look Once (YOLO), U-Net, Mask R-CNN, and Single Shot Detection (SSD), will be helpful for semantic segmentation. Since firm-applicable AI devices and mobile and web applications are in demand, the dataset collected in this study will offer multiple options for integrating ML models into AI devices. In countries with weather and climate similar to Bangladesh, data scientists may use their dataset in that context.

Specifications Table

**Table:** 

Subject	Agriculture Engineering, Computer Vision, Image Processing, IoT-based smart agricultural solutions, and Pattern Recognition.
Specific subject area	Deep learning-based image detection and classification of plant leaf disease. (YOLO, SSD, Vision Transformer, U-Net)
Data format	Raw: jpg Annotation: XML and txt
Type of data	Image
Data Collection	2159 papaya leaf images were collected, encompassing four papaya leaf diseases and healthy leaves. All the pictures were captured using two devices with high-resolution cameras, Oppo and Redmi. The original images were in JPG format, featuring dimensions of 3468×4624 pixels and 3000×4000 pixels, with a resolution of 72 dpi. Then, they were rescaled to the dimension of 640×480 pixels with a resolution of 96 dpi. The photographs were captured in the Changao area of Ashulia, Dhaka, Bangladesh, from July 12 to August 2, 2023, at coordinates 23° 53′ 2″ N and 90° 19′ 28″ E. With the help of a plant pathology specialist, the images were subsequently classified into 5 classes: *Anthracnose, Bacterial spot, Curl, Ring spot,* and *Healthy*. The photos were manually annotated with information about the disease category and location, and the annotations were saved in XML and TXT forms for usage with various models.
Data Source Location	City: Changao, Ashulia, Dhaka Country: Bangladesh Coordinates: 23° 53′ 2″ N and 90° 19′ 28″ E
Data accessibility	Repository name: Mendeley Data Data identification number: 10.17632/p997fvf526.1 Direct URL to data: https://data.mendeley.com/datasets/p997fvf526/2

## 1 Value of the Data

• Data is essential for machine learning applications [1], [2], [3], [4], [5], [6], [7], [8], [9]. This dataset offers a collection of images of healthy and diseased papaya leaves. It provides a comprehensive resource for researchers and developers in data science. It enables detailed study and comparison across different types of diseases. The dataset might be valuable for more accurate diagnosis, advanced computer-aided plant disease diagnosis, and understanding of disease patterns.
• The dataset consists of high-resolution images with detailed annotations. This quality can ensure that machine learning models trained on this dataset can achieve higher accuracy, as precise annotations help effectively learn disease characteristics.
• This dataset can effectively assist agricultural scientists and plant pathologists working on disease management strategies. By utilizing this dataset, data scientists and industry experts can find solutions to aid farmers in making informed decisions about disease prevention.
• This dataset is publicly available for open research and collaboration among the scientific community. Researchers from different institutions can access and utilize the dataset, fostering collaborative efforts in combating papaya leaf diseases globally.
• This dataset can be used as a practical resource in education and research. It has practical applications. For example, mobile phone-based machine learning models can be implemented using the dataset. It can also provide students and educators using real-world data for hands-on experience in image processing, machine learning, and agricultural studies.
• Rashid et al. [10] have contributed to the research community by providing a dataset on Papaya Leaf diseases in Bangladesh. This dataset includes common diseases such as Leaf Curl, Papaya Mosaic, Ring Spot, and Mites or Mealybug disease. It is currently the only available dataset comprehensively covering these common Bangladeshi Papaya diseases. This dataset contains a total of 1400 original images. However, this dataset presents 2159 original images and annotated data with additional diseases, namely Anthracnose and Bacterial Spot, which were not included in the currently available dataset. This expanded dataset will provide a more comprehensive resource for researchers.

## 2 Background

This dataset was created for research purposes, focusing on Bangladeshʼs agricultural industry. Papaya is a popular fruit and vegetable in Bangladesh, but leaf diseases often threaten harvesting. This dataset aims to develop a deep learning model that can detect these diseases through an Android application, allowing farmers to identify them more accurately and at a lower cost. To achieve this, a research experiment was conducted using YOLOv8 and Vision Transformer algorithms. The images were annotated to enable algorithms like YOLOv8 to detect the diseases, while Vision Transformer used direct images for the experiment. This dataset aims to build a robust method for detecting and classifying papaya leaf diseases (Table 1).

**Table 1:** Brief description of the data collection.

No.	Particulars	Description
1	Leaf	Papaya
2	Number of diseases considered	5 (Healthy Included)
3	Photo Time	• July 12–August 2,2023 shooting every day. • The times are all cloudy and sunny in both daytime hours.
4	Geographical Location	23° 53′ 2″ N 90° 19′ 28″ E
5	Climatic	Sunny and Rainy both.
6	Temperature	30 °C (Average)

## 3 Data Description

The “BDPapayaLeaf” dataset is a collection of pictures taken at several gardens in Changao, Ashulia, and Dhaka, Bangladesh. There are 2159 original images in the dataset. It represents the proposed datasetʼs directory tree. The leading directory is designated as “BDPapayaLeaf”. It has two subdirectories: one for photos of raw leaves that were first gathered and converted to modified pixel format with five different classes and the other for images that have undergone processes like annotation. TXT and XML are the two formats in the annotation directory (Table 2).

**Table 2:** Description of the dataset for leaf disease of papaya (raw image).

Class	Anthracnose	Bacterial spot	Curl Leaf	Ring spot	Healthy
Num. of images	355	458	585	533	228

### 3.1 Disease description

The following sections describe the diseases:

#### 3.1.1 Anthracnose

**Fig. 1:**
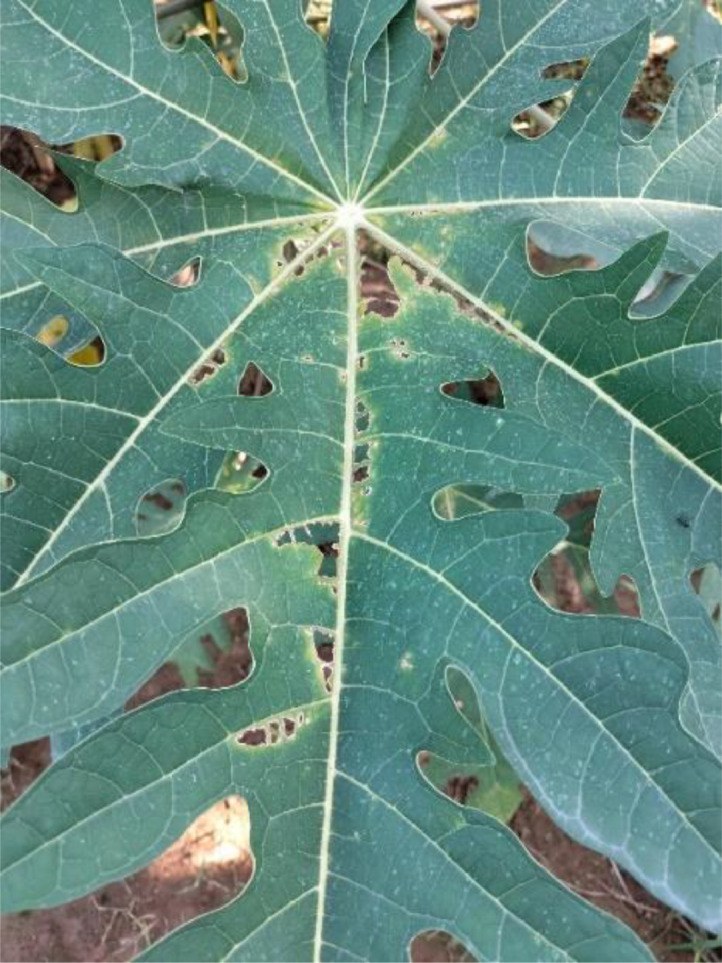
The Anthracnose disease of papaya leaf.

The fungus anthracnose damages the leaves, fruits, and stems of papaya plants, among other parts. The fungus ‘Colletotrichum gloeosporioides’ is the cause of this disease [11]. The anthracnose effects and symptoms are highest in areas with warm, rainy temperatures. Dark, deep blisters on leaves with a possible yellow ring are signs of anthracnose. After harming the leaves, it eventually affects the fruits as tiny, dark spots that grow larger and collapse, causing fruit rot. Severe leaf infections might result in early fruit drop and decreased output. The disease is spread via wind, contaminated tools, and rain splashes. Fig. 1 shows an early stage of anthracnose (see Fig. 1) (Table 3).

**Table 3:** Description of the dataset for leaf disease of papaya (annotation).

Class	Anthracnose	Bacterial spot	Curl Leaf	Ring spot	Healthy
Num. of images	210	210	210	210	210

#### 3.1.2 Bacterial spot

**Fig. 2:**
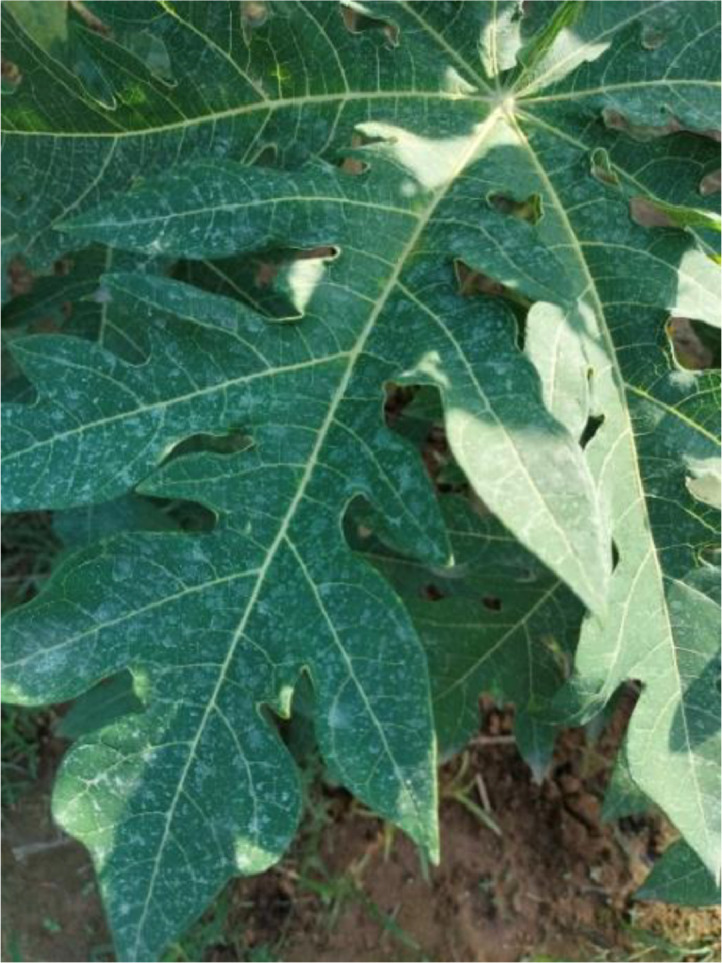
The bacterial spot disease of papaya leaf.

Xanthomonas campestris pv. Caricae is a common cause of bacterial illness and is known as a bacterial spot” in papayas. It mainly affects papaya plant leaves and can significantly reduce production. Small, water-soaked blisters on leaves that are the primary sign of the bacterial spot on the leaf and eventually become brown or black with a yellow ring shape are signs of bacterial spots. These spots may combine, causing peeling and leaf rot. This effect can not only affect the leaves; fruits with the infection may also have elevated, corky pimples. Rain, wind, and contaminated instruments spread bacterial spots from leaves to fruit (see Fig. 2).

#### 3.1.3 Curl

**Fig. 3:**
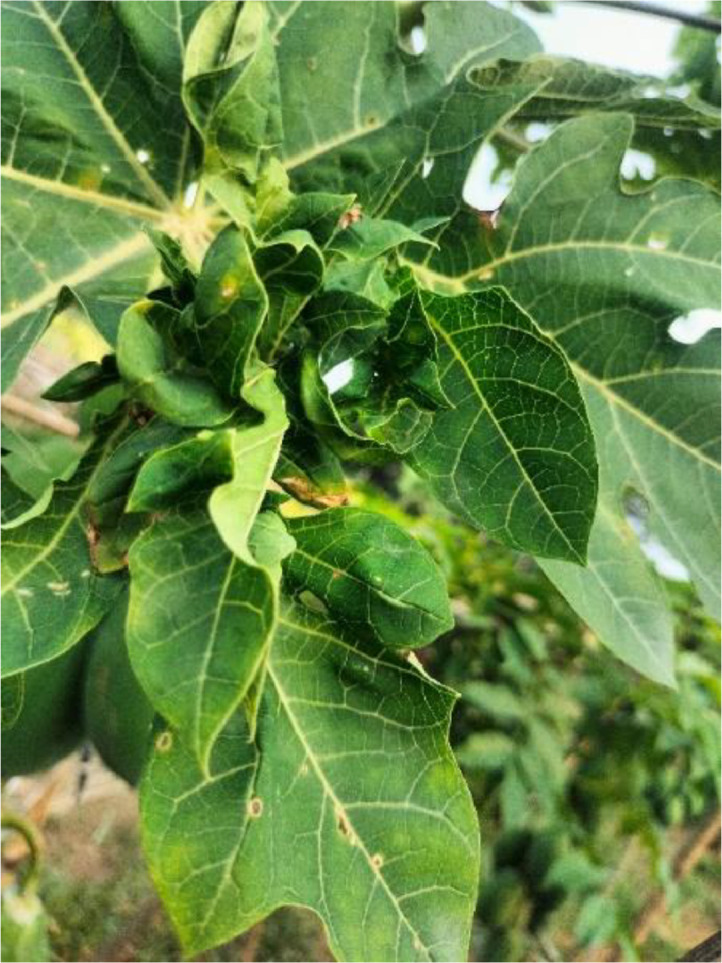
The of Curl disease papaya leaf.

Papaya leaf curl disease, known as (PaLCuD) is caused by the papaya leaf curl virus (PaLCuV) [12]. The whitefly Bemisia tabaci Genn may spread the disease. Whiteflies feed on infected papaya plants and then transmit the virus to healthy plants as they feed. A geminivirus was suspected of being involved in this disease, too. There are different symptoms of this disease, and the most important symptoms are the downward curving and cupping of the leaves, followed by vascular transparency and thickening up. These disease-affected leaves become fragile and complex, and their petals twist zigzag-fashion. Diseased plants may produce a small number of cracked, suddenly falling fruits [13]. It impacts production and the growth of plants, fruit size, and quality and has a significant economic impact on Papaya panting (see Fig. 3).

#### 3.1.4 Ring spot

**Fig. 4:**
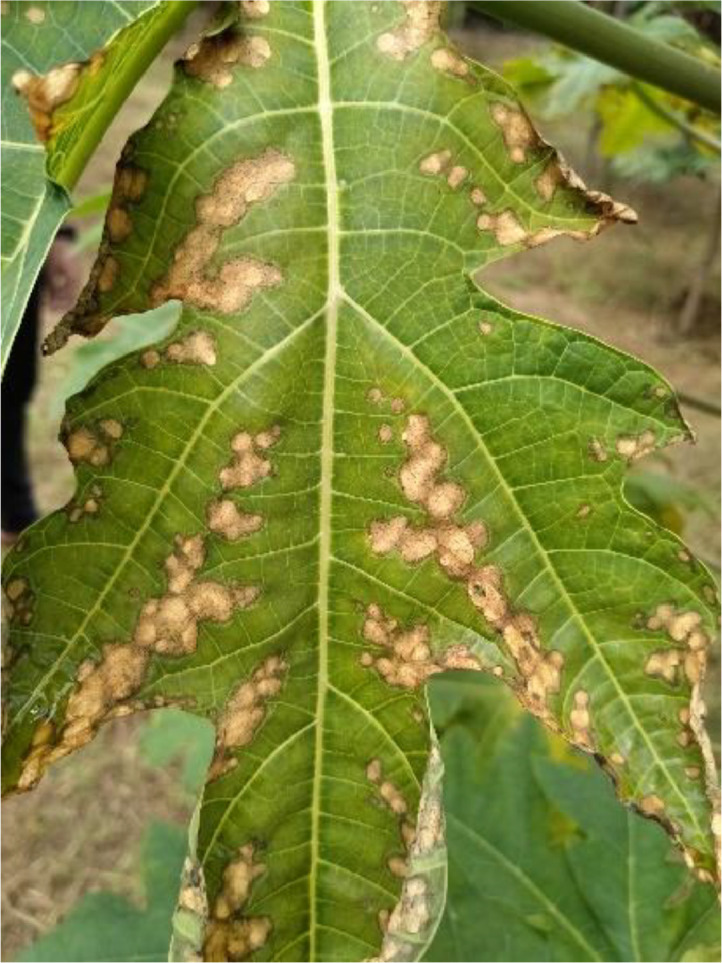
The ring spot disease of papaya leaf.

Viruses and parasites are just two of the many agents that can cause papaya ring spot leaf disease. As seen in Fig. 4, this disease manifests as a yellow right-shaped shape on ripe papaya leaves. Ring-like infections on leaves, which may also be accompanied by leaf bending and yellowing, are among the many symptoms of ring spots. The ring spot virus spreads quickly through the plant's branches and main stem, with illnesses often showing up 13 or 14 days after infection on adult leaves in the final stage of the canopy (Jensen et al., 1949). Fruit yield and quality may suffer because of this disease. Key preventive strategies include limiting the disease's spread and parasite vector control (see Fig. 4).

#### 3.1.5 Healthy

**Fig. 5:**
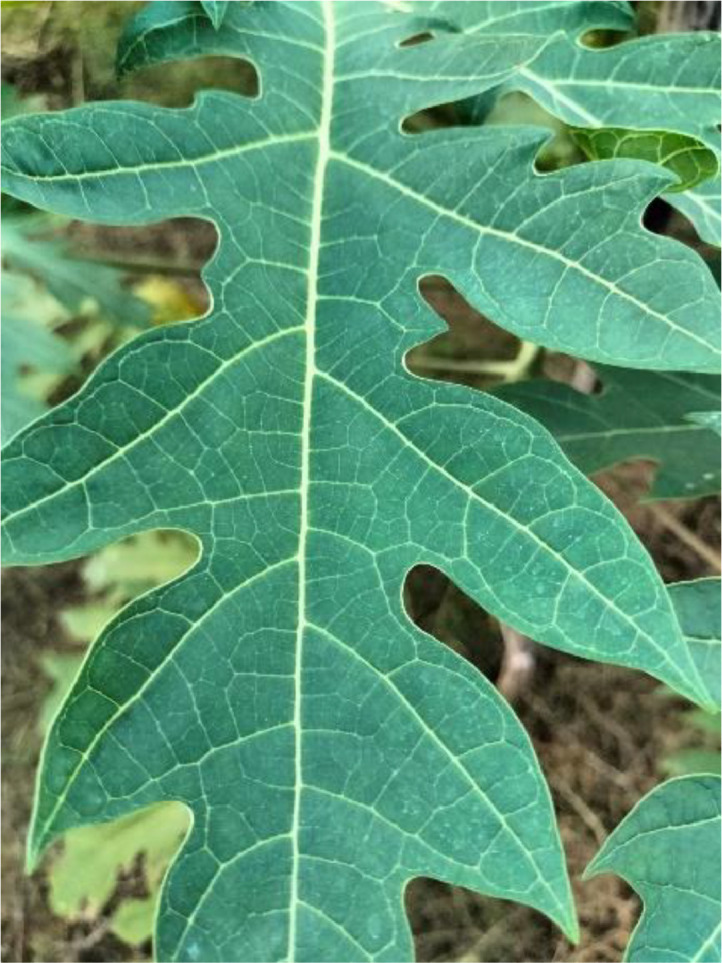
The healthy papaya leaf.

Healthy leaves are disease-free leaves. However, due to many viruses, pests, and bacterial infections, healthy leaves turn into diseased leaves. Healthy leaves on a papaya tree indicate a healthy papaya fruit. Because of an unhealthy leaf, other healthy leaves and fruit can also be affected. Immature fruit falling, not being correctly ripe, rotten after ripe, and not gaining the proper shape of the fruit are the causes of disease-affected leaves. Due to disease, farmers can face a substantial financial loss (see Fig. 5).

### 3.2 Significance of the Dataset

**Fig. 6:**
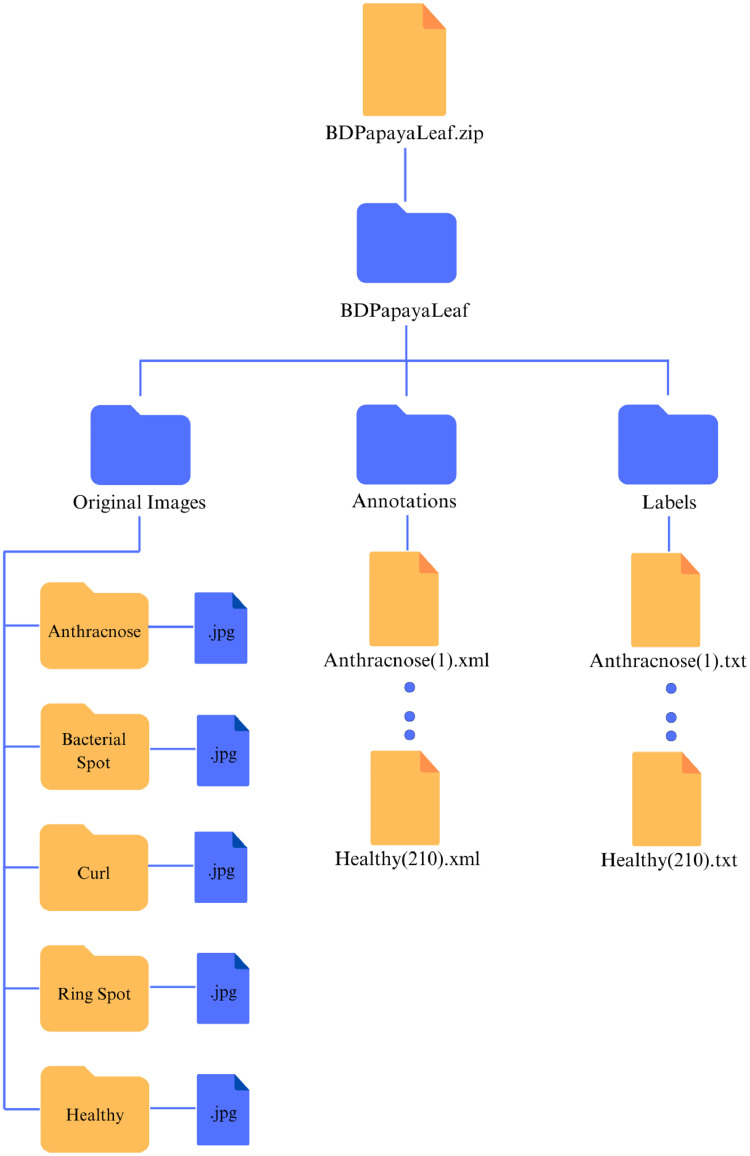
Folder hierarchy tree.

The papaya disease costs many growers a tremendous deal of money each year. Farmers are unaware of the disease-mitigation techniqueʼs detecting strategy. They only learned about the infections after the papaya had already been sick and wasted. Because of the loss of cultivation, many are reluctant to begin planting papayas. Over the past few years, thousands of researchers have concentrated on the papaya disease recognition system to reduce farmer losses [14]. We have included five important papaya fruit leaf diseases from Bangladesh in our dataset to help the researchers. Thanks to this dataset, which can provide a new dimension to papaya leaf disease detection and classification, the researcher can create new models and algorithms to help farmers identify and classify papaya leaves before the disease spreads to the entire garden. Early diagnosis of papaya leaf disease can prevent farmers from experiencing significant financial loss since they can recognize the condition and take the required steps to eliminate it as quickly as possible (Fig. 6).

Our dataset has been manually annotated to provide four practical diseases, with one healthy categorization dataset used as a resource for computer scientists working on scholarly research and model construction. It also benefits those in the agricultural industry by providing real-world papaya leaf disease images that they can use to study and contrast with healthy leaves. Ultimately, this dataset can offer helpful cultivation advice for papaya fruit producers for different leaf illnesses, assisting them in strengthening their management for disease detection and cultivation cure procedures. Computer scientists conducted research and development to create this dataset. In the real world, this dataset's contribution is rather considerable (Table 4).

**Table 4:** Brief description of the dataset.

No	Particulars	Description
1	Leaf	Papaya
2	Number of growing periods considered	5
3	Original Image	JPG; 3468×4624 pixels and 3000×4000 pixels; 72 dpi
4	Converted Image	640×480 pixels; 96 dpi
5	Annotation file format	XML, TXT
6	Data set size	Size of each image: 61–229 KB Original Image folder size: 231 MB Annotations folder size: 1.97 MB Labels folder size: 281 KB

### 3.3 Description of the dataset folder

The root directory contains one file: BDPayapaLeaf.zip. The file extension .zip is a zip folder. The zip folder contains a dataset folder, BDPayapaLeaf. We have created a zip folder, which is comparatively more straightforward to download. The dataset folder contains all the other subfolders of the dataset. The BDPayapaLeaf dataset folder contains three subfolders: Original Images, Annotations, and Labels. The description of the folder is explained in Table 5.

**Table 5:** Brief description of the folders.

No	Folder name	File format in the folder
1	BDPayapaLeaf.zip	zip (itself)
2	BDPayapaLeaf	All format together
3	Original Images	jpg
4	Annotations	XML
5	Labels	txt

#### 3.3.1 Original images

The Original Images folder contains all the images in the dataset. The folder size is 231 MB. The highest image size of the image is 229 KB, and the lowest is 61 KB. All the images were reshaped into 640×480-pixel format, and the resolution was also increased from 72 dpi to 96 dpi through the software ‘Fast Stone Photo Resizer’. This file has five subfolders: anthracnose, bacterial spot, curl, ring spot, and healthy. Each folder contains images of those classes individually. We have made the images in this structure due to classification help. Researchers can easily find the classified images in the Original Image folder and load them into their model to train their papaya leaf disease classification. The images were renamed into each class, such as Anthracnose(1).jpg, Anthracnose(355).jpg, and BacterialSpot(1).jpg. …… BacterialSpot(458).jpg, Curl(1).jpg ……..Curl(585).jpg, RingSpot(1).jpg … RingSpot(533).jpg, Healthy(1).jpg ………Healthy(228).jpg.

#### 3.3.2 Annotations

**Fig. 7:**
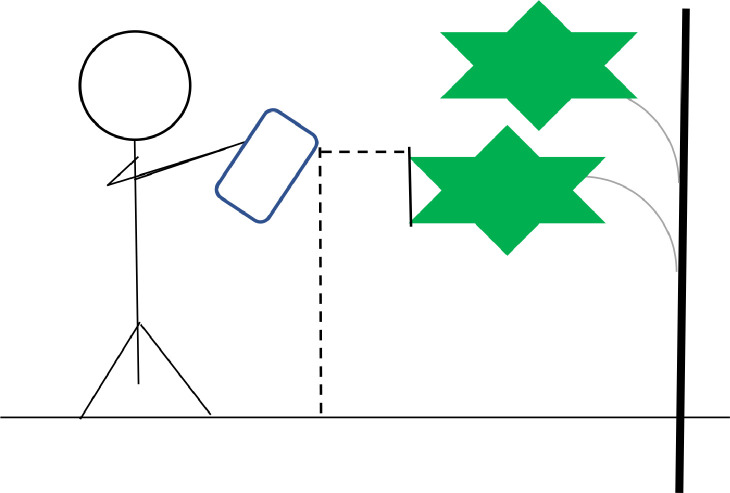
Photo capturing method.

This folder contains 1050 XML files. This is the annotation folder. The folder size is 1.97 MB. XML files have the same naming style as the original image folder without classification. However, anyone can classify those XML files into separate folders, if needed, according to the name of the XML files. This is the reason we have renamed the files using the format mentioned. The annotation includes the class names of the diseases individually (Fig. 7).

#### 3.3.3 Labels

This folder was created by the researcher working with the YOLO model. The YOLO model mainly works with annotation files in text format. The file size is 281 KB. This folder also contains 1050 files. Four disease classes and one healthy class are categorized as 0–4 integer numbers (Anthracnose:0, BacterialSpot:1, Curl:2, RingSpot:3, and Healthy:4). This folder has the same name style as the original folder, which can be classified according to researchers’ needs.

## 4 Experimental Design, Materials and Methods

### 4.1 Theoretical knowledge gathering

Before executing our dataset collection methodology, we studied the papaya tree and its leaves. We got help from online blogs and posts about papaya trees, papaya leaves, papaya fruit diseases, papaya leaf diseases, how to detect those diseases, symptoms of the diseases, causes of those diseases, etc. Our knowledge-gathering session's sources were agricultural sites and previous research on papaya leaves. This is essential for researchers with enough knowledge to collect data about their research topic.

### 4.2 Leaf selection

Leaf selection is the most important part after the knowledge-gathering session of a dataset related to leaf diseases, either detection or classification. Among several trees, choosing a tree of reachable height is essential, as is having a tree that has enough leaves to represent all the diseases we wanted to cover in our dataset. Leaves were chosen randomly from the papaya trees. In our dataset, it is notable that all the leaves we have taken are mature. Papaya leaf diseases hit the leaves when they turn into mature leaves. So, we have ensured that all the disease-affected mature leaves could be collected.

### 4.3 Digital image acquisition

The image acquisition process should always be done correctly. Because clear and perfect pictures will increase the accuracy of disease detection, identification, and classification. If the images are not taken clearly, it can cause a wrong identification and classification that will cause a missed interpretation. The accuracy of the models is also determined by how clearly and precisely the photographs are taken. Because if the dataset is not precise enough, models cannot provide a correct accuracy score. So, we focused on this section during the image collection (Table 6).

**Table 6:** Description of the camera device.

Particulars	Device name	
	OPPO	Redmi
Camera manufacturer	OPPO	Xiaomi
Camera model	OPPO F21s PRO	Xiaomi Redmi Note 11 pro
Camera pixel	64 MP,2MP,2MP	108 MP,8MP,2MP
Aperture Value	f/1.8	f/1.9
Exposure time	1/472 s.	–
Camera flash	None	None
Monitoring range	Size: 3468×4624	Size: 3000×4000
Imaging	Resolution: 72 dpi Bit Depth:24	Resolution: 72 dpi Bit Depth:24

We initially used two devices, ‘OPPO’ and ‘Redmi’, to capture all the pictures. The ‘Oppo’ device captured an image size of 3468×4624 pixels with a 72 dpi resolution, and the Redmi device captured images in a 3000×4000 image size with a 72 dpi resolution. Initially, images were taken in jpg format.

• The camera angle was 45° according to the ground. This angle provides a clear view of the leafʼs surface, which highlights the details such as texture, color, and any disease symptoms.
• The device position was 4.5 feet from the ground (depending on the vertical leaf position from the ground) (vertical dotted line) since the papaya tree leaves were, on average, the same height. Keeping the camera at 4.5 feet above the ground ensures all images have the same perspective, making them comparable and reliable for analysis.
• The device distance from the target leaf was 50–60 cm (horizontal dotted line). This distance allows for capturing fine details without distortion and for identifying slight disease symptoms or features.

### 4.4 Leaf classification

**Fig. 8:**
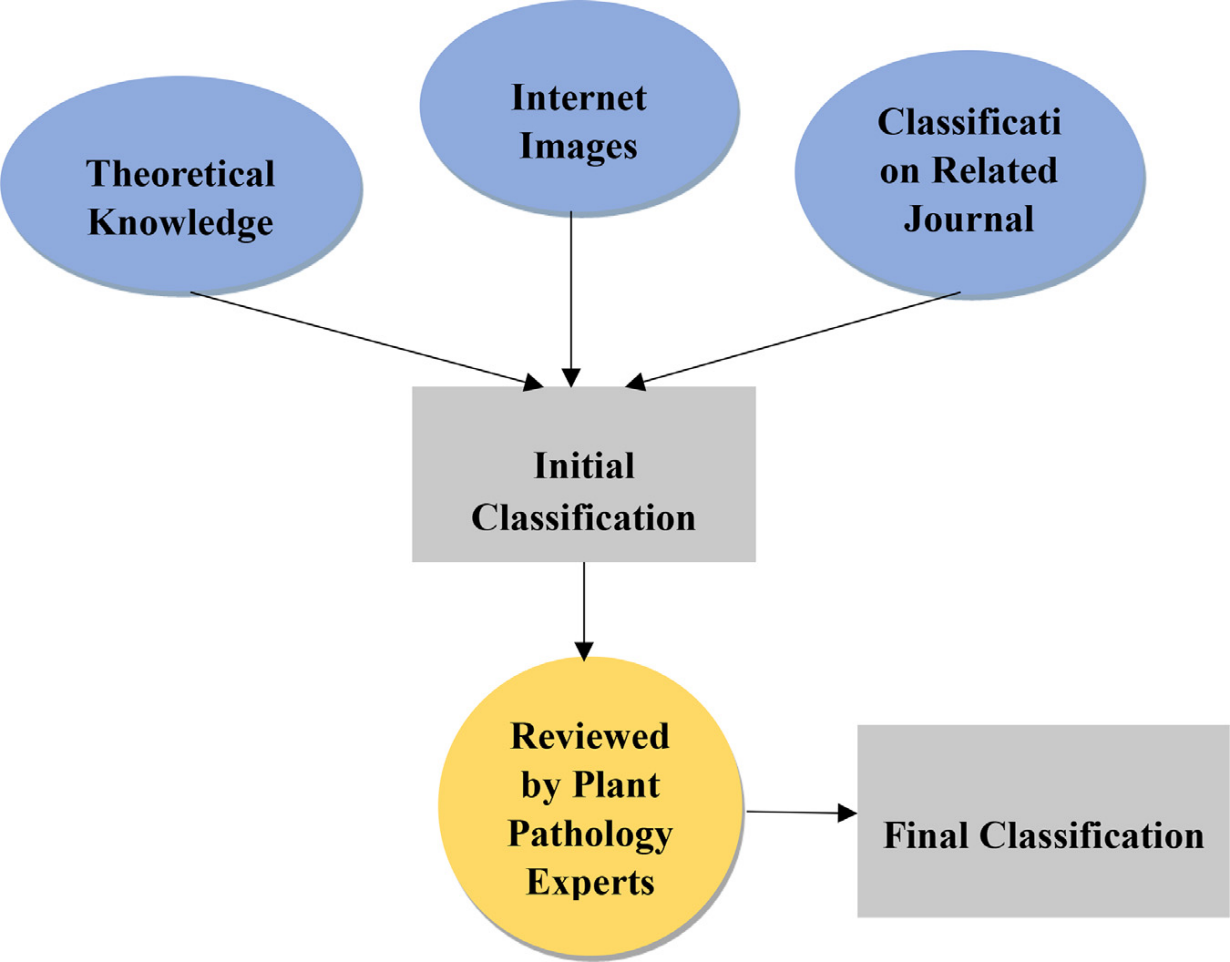
Leaf diseases classification criteria.

Classifying the collected leaves according to their disease ensures the correctness of the disease classification model. Diseases are classified into ‘Anthracnose’, ‘Bacterial spot’, ‘Curl’, ‘Ring spot’ and the ‘Healthy’ class. This part was done by collaborating with a plant pathology expert and the theoretical knowledge we gathered before executing the data collection method. The whole classification criteria are explained in Fig. 8.

### 4.5 Image processing

Raw images were processed by height and width-reducing pixels. High-resolution photos were decreased into low-resolution images to compress the file size, which will be easy for the researchers to use in their model. An application software called ‘Fast Stone Photo Resizer’ has been used to resize the pictures and increase their resolution. While another online image processing tool provides image processing access to a certain number of images, this software helps to process a large number of images together. The resized image size was 640×480 pixels, and the resolution was 96 dpi. Image processing information is provided in the previous section.

### 4.6 Image annotation

Image annotation was the last step of our methodology. The image annotation process involves labelling images with class labels and drawing bounding boxes around objects of interest. This creates structured data that enables supervised learning for object detection models. According to the information (e.g. height and width) in the bounding box and for each specific class, numerical values are assigned and stored in Txt and XML file format. Image annotation is mandatory for specific deep learning models (such as YOLO, R-CNN, SSD, U-Net, and FCN). From each class, we have taken 210 images for annotation. All the annotation files are in XML and Txt formats.

The annotation process is conducted using a tool called ‘Make Sense AI’. This tool provides a reliable interface for image annotation that is faster than other tools (e.g., Roboflow).

Since disease areas in the leaf's annotation require domain expert knowledge, two agriculture engineers and one farmer were invited to assist in identifying the disease. Their suggestions were taken while annotations were conducted. They also extended their help in classifying and labelling the collected images.

## Limitations

This dataset's limitation is that not all the images are thoroughly annotated. We would annotate all images further. Annotations from three different experts will further improve the dataset.

## Ethics Statement

Neither plants nor animals contracted an infection while the data were being gathered. The current work does not use human subjects, animal trials, or data gathered from social media sites. The authors strictly maintained the ethical code of data in brief during the dataset collection experiment.

## CRediT Author Statement

**Sumaya Mustofa:** Writing - Original Draft, Visualization, Data Curation; **Md Taimur Ahad:** Supervision, Writing - Review & Editing; **Yousuf Rayhan Emon**: Review & Editing; **Arpita Sarker:** Conceptualization, Investigation.

## Data Availability

BDPapayaLeaf: A annotation based image dataset of papaya leaf disease (Original data) (Mendeley Data).

## Appendix Supplementary materials

Image, application 1
