# Supplementary figures and images for: BDPapayaLeaf: A dataset of papaya leaf for disease detection, classification, and analysis
Source: Data Brief. 2024 Sep 10;57:110910. doi: 10.1016/j.dib.2024.110910 (PMC11460515; doi:10.1016/j.dib.2024.110910)

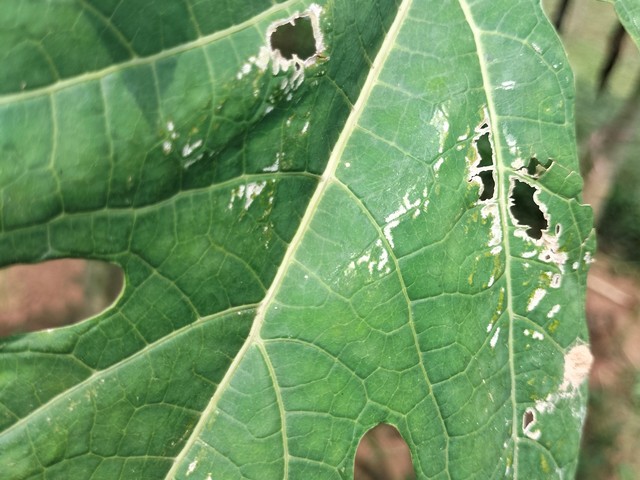

Supplement: Supplementary file 1 [file mmc1.zip › Demo dataset/Original Images/Anthracnose/Anthracnose(1).jpg]

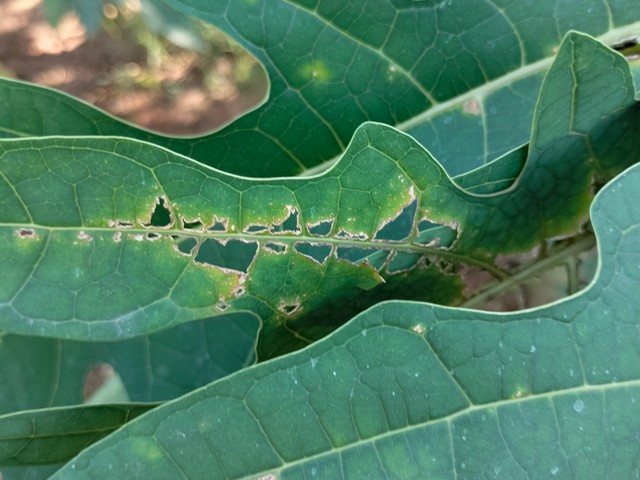

Supplement: Supplementary file 1 [file mmc1.zip › Demo dataset/Original Images/Anthracnose/Anthracnose(10).jpg]

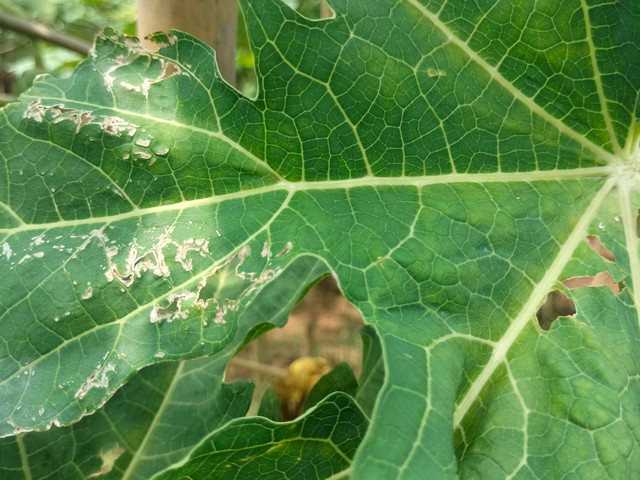

Supplement: Supplementary file 1 [file mmc1.zip › Demo dataset/Original Images/Anthracnose/Anthracnose(2).jpg]

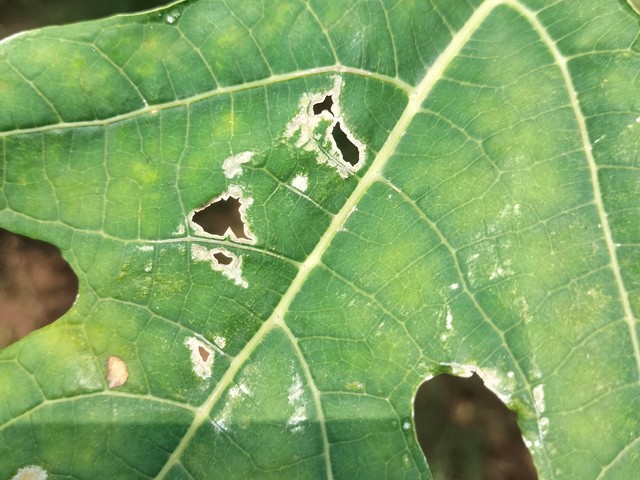

Supplement: Supplementary file 1 [file mmc1.zip › Demo dataset/Original Images/Anthracnose/Anthracnose(3).jpg]

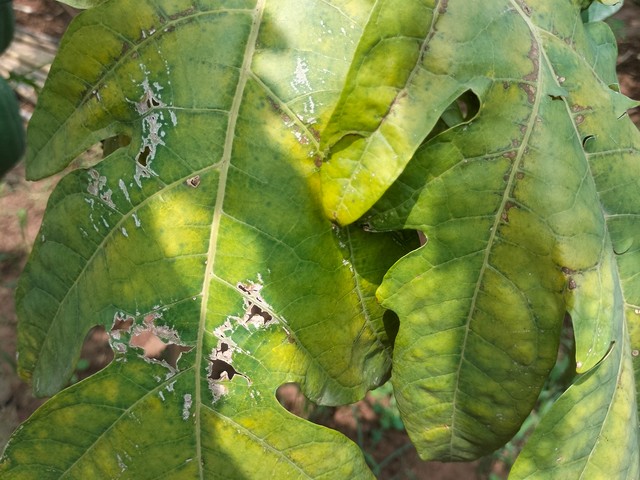

Supplement: Supplementary file 1 [file mmc1.zip › Demo dataset/Original Images/Anthracnose/Anthracnose(4).jpg]

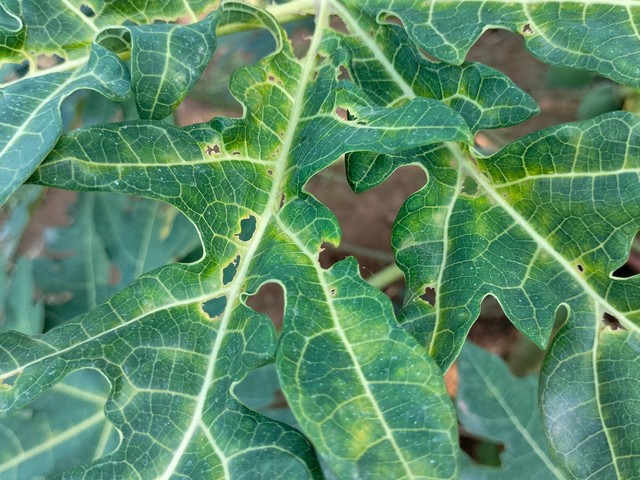

Supplement: Supplementary file 1 [file mmc1.zip › Demo dataset/Original Images/Anthracnose/Anthracnose(5).jpg]

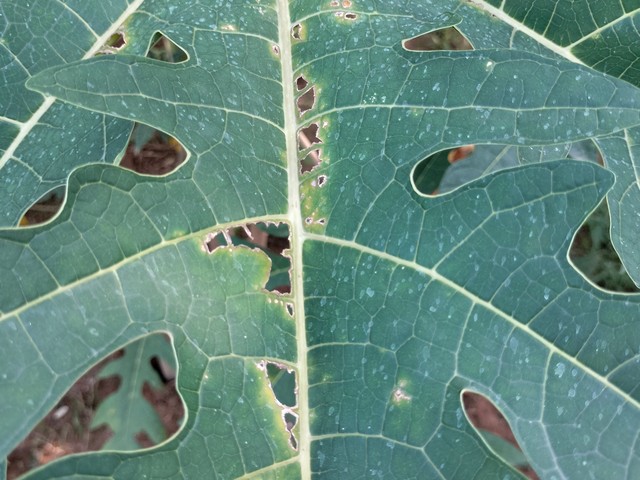

Supplement: Supplementary file 1 [file mmc1.zip › Demo dataset/Original Images/Anthracnose/Anthracnose(6).jpg]

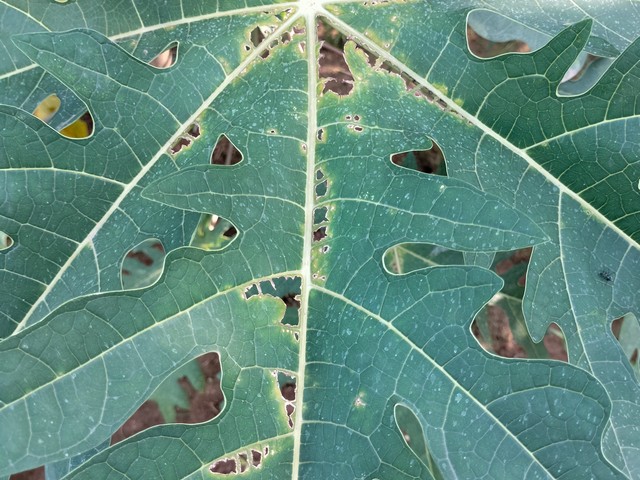

Supplement: Supplementary file 1 [file mmc1.zip › Demo dataset/Original Images/Anthracnose/Anthracnose(7).jpg]

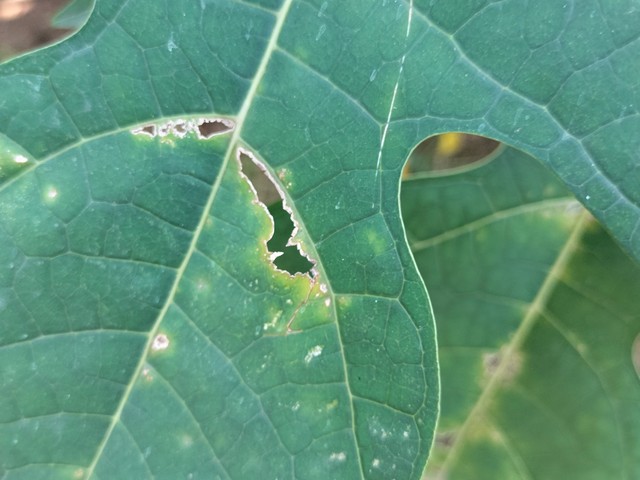

Supplement: Supplementary file 1 [file mmc1.zip › Demo dataset/Original Images/Anthracnose/Anthracnose(8).jpg]

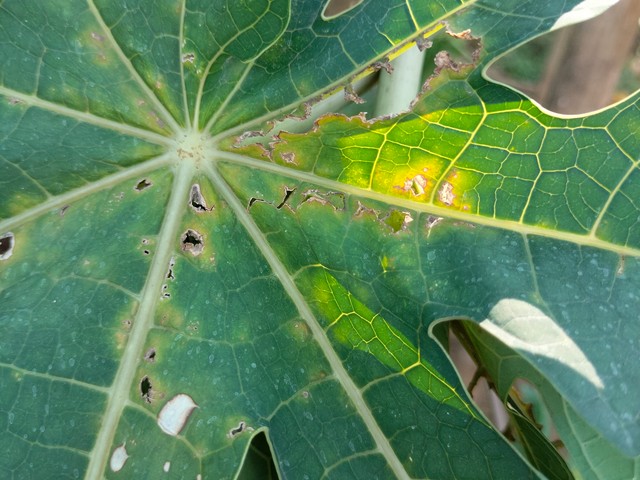

Supplement: Supplementary file 1 [file mmc1.zip › Demo dataset/Original Images/Anthracnose/Anthracnose(9).jpg]

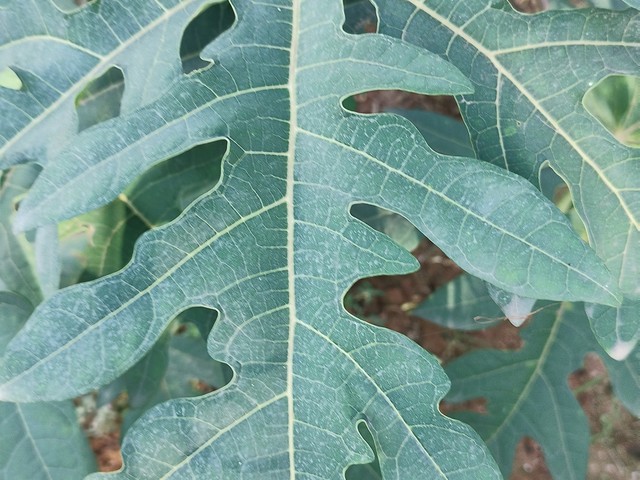

Supplement: Supplementary file 1 [file mmc1.zip › Demo dataset/Original Images/BacterialSpot/BacterialSpot(1).jpg]

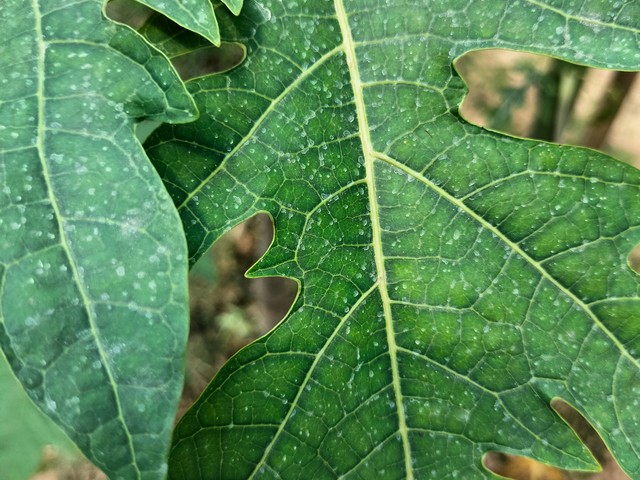

Supplement: Supplementary file 1 [file mmc1.zip › Demo dataset/Original Images/BacterialSpot/BacterialSpot(10).jpg]

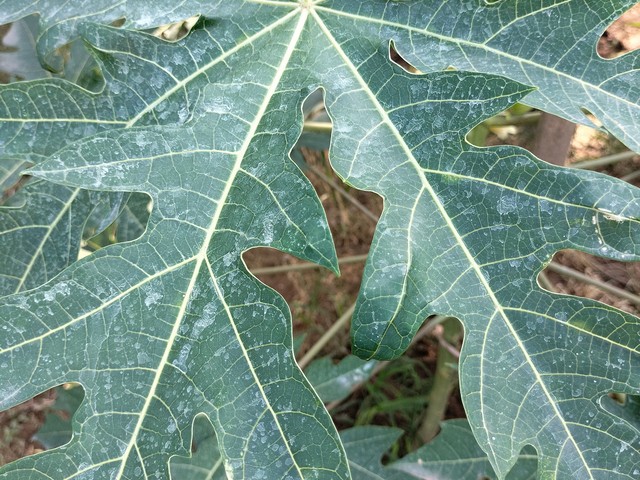

Supplement: Supplementary file 1 [file mmc1.zip › Demo dataset/Original Images/BacterialSpot/BacterialSpot(2).jpg]

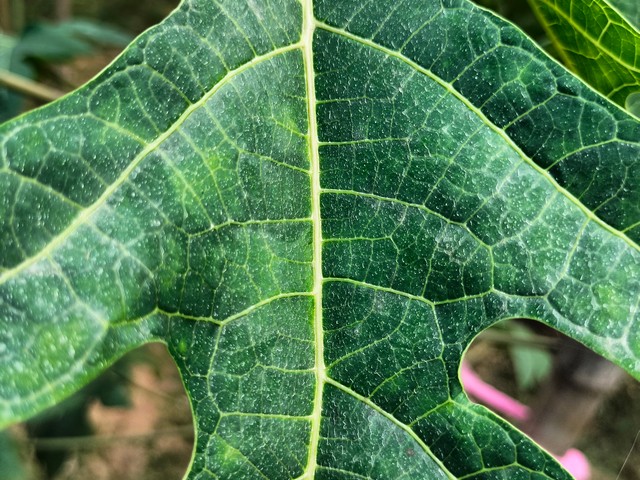

Supplement: Supplementary file 1 [file mmc1.zip › Demo dataset/Original Images/BacterialSpot/BacterialSpot(3).jpg]

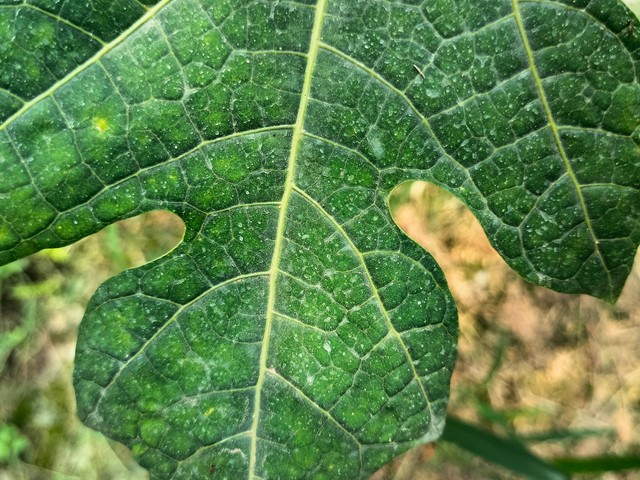

Supplement: Supplementary file 1 [file mmc1.zip › Demo dataset/Original Images/BacterialSpot/BacterialSpot(4).jpg]

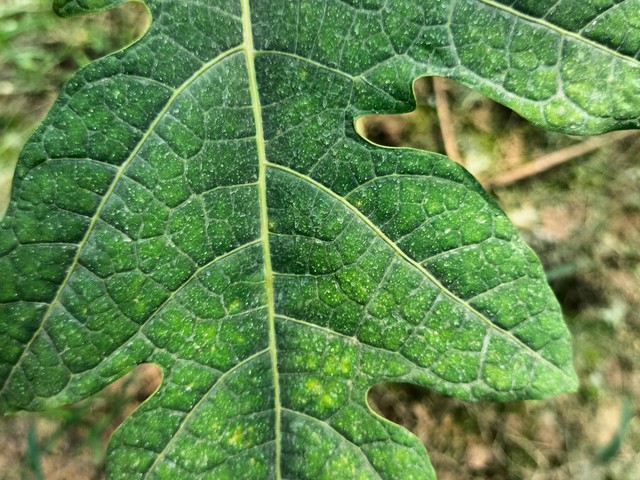

Supplement: Supplementary file 1 [file mmc1.zip › Demo dataset/Original Images/BacterialSpot/BacterialSpot(5).jpg]

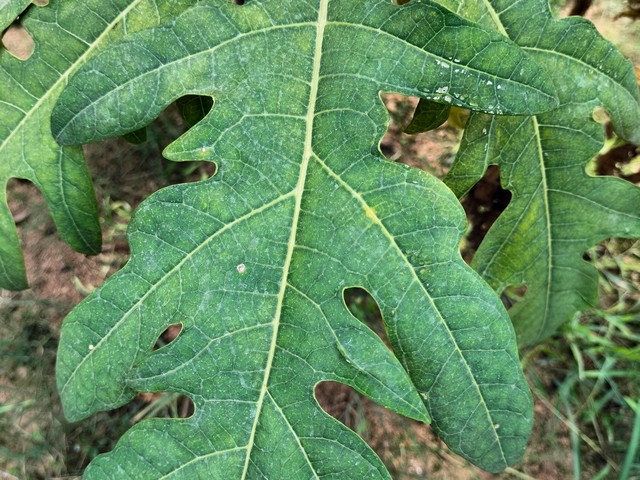

Supplement: Supplementary file 1 [file mmc1.zip › Demo dataset/Original Images/BacterialSpot/BacterialSpot(6).jpg]

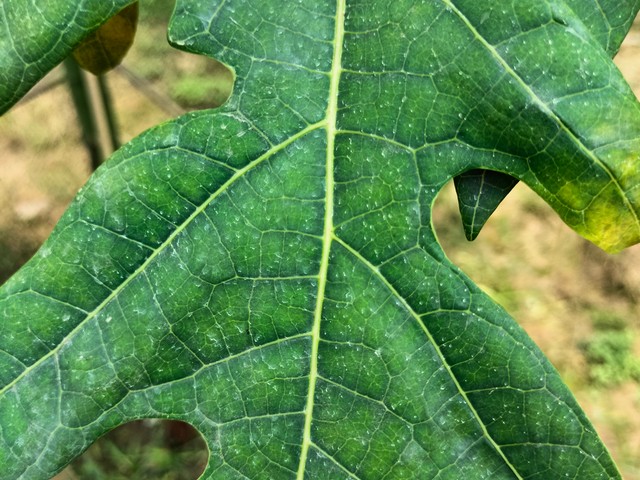

Supplement: Supplementary file 1 [file mmc1.zip › Demo dataset/Original Images/BacterialSpot/BacterialSpot(7).jpg]

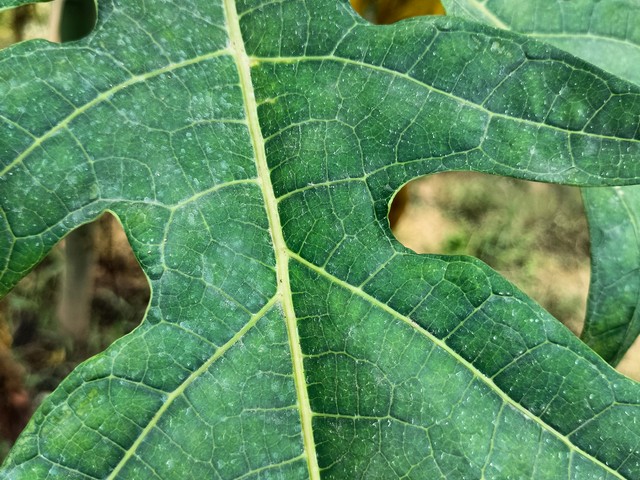

Supplement: Supplementary file 1 [file mmc1.zip › Demo dataset/Original Images/BacterialSpot/BacterialSpot(8).jpg]

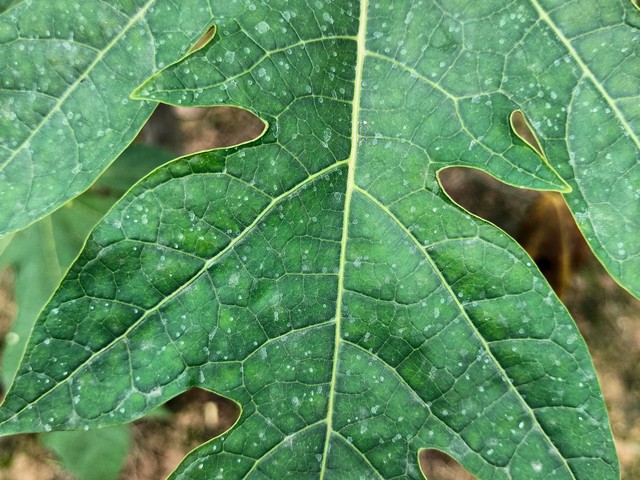

Supplement: Supplementary file 1 [file mmc1.zip › Demo dataset/Original Images/BacterialSpot/BacterialSpot(9).jpg]

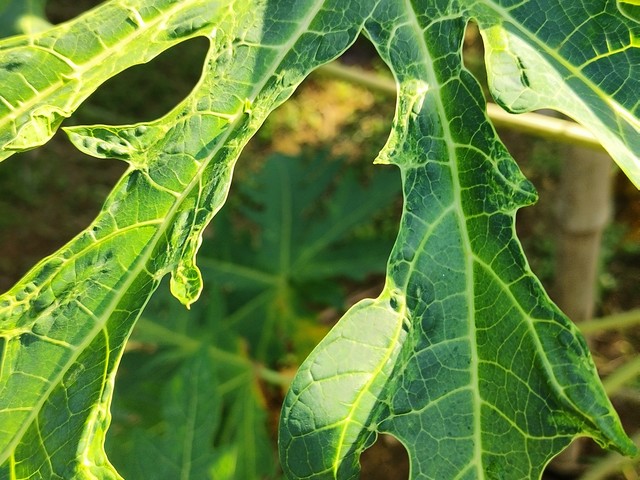

Supplement: Supplementary file 1 [file mmc1.zip › Demo dataset/Original Images/Curl/Curl(1).jpg]

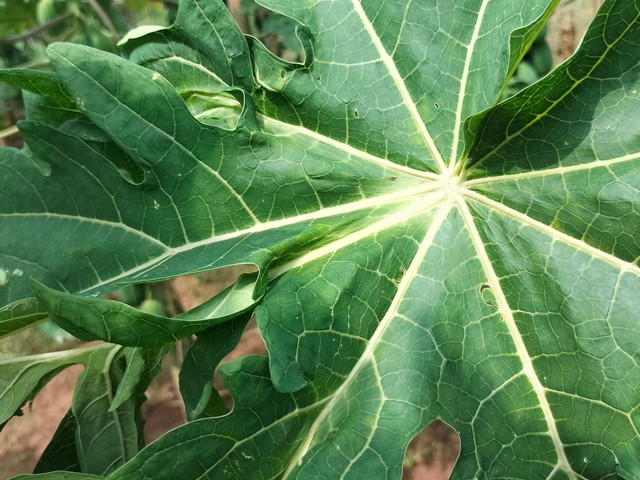

Supplement: Supplementary file 1 [file mmc1.zip › Demo dataset/Original Images/Curl/Curl(10).jpg]

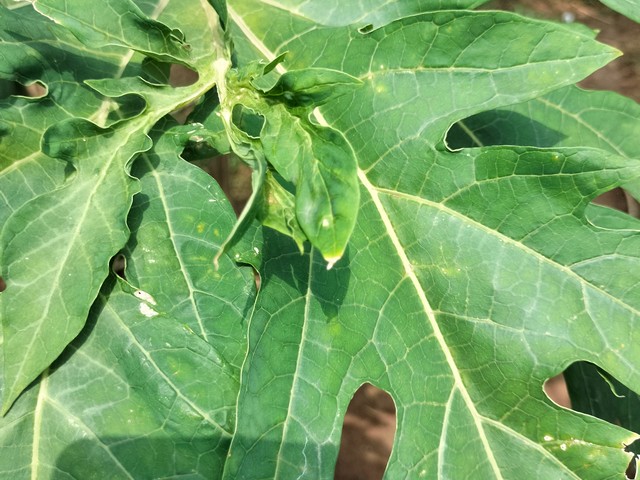

Supplement: Supplementary file 1 [file mmc1.zip › Demo dataset/Original Images/Curl/Curl(2).jpg]

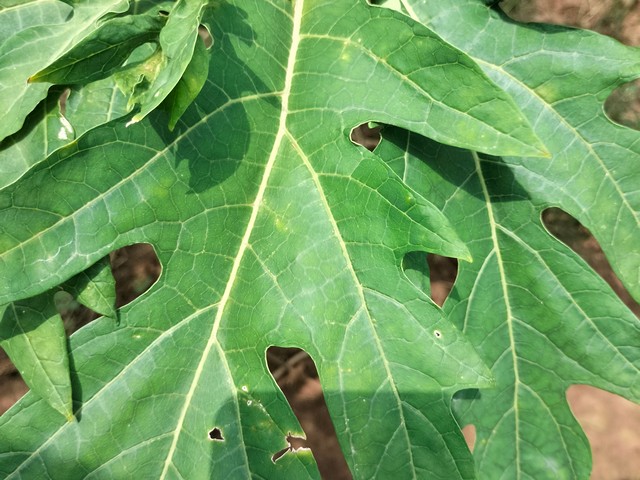

Supplement: Supplementary file 1 [file mmc1.zip › Demo dataset/Original Images/Curl/Curl(3).jpg]

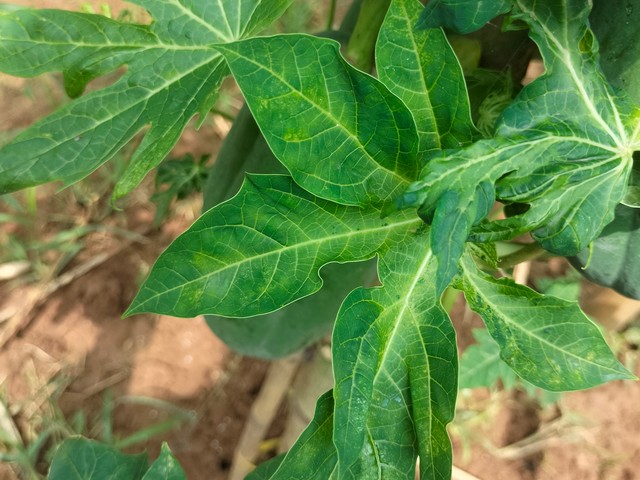

Supplement: Supplementary file 1 [file mmc1.zip › Demo dataset/Original Images/Curl/Curl(4).jpg]

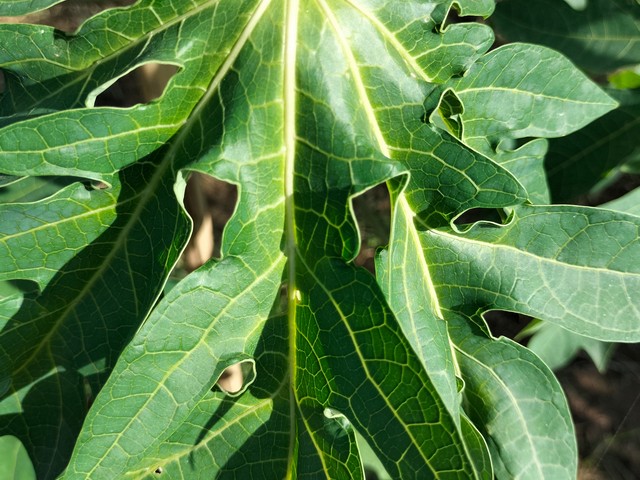

Supplement: Supplementary file 1 [file mmc1.zip › Demo dataset/Original Images/Curl/Curl(5).jpg]

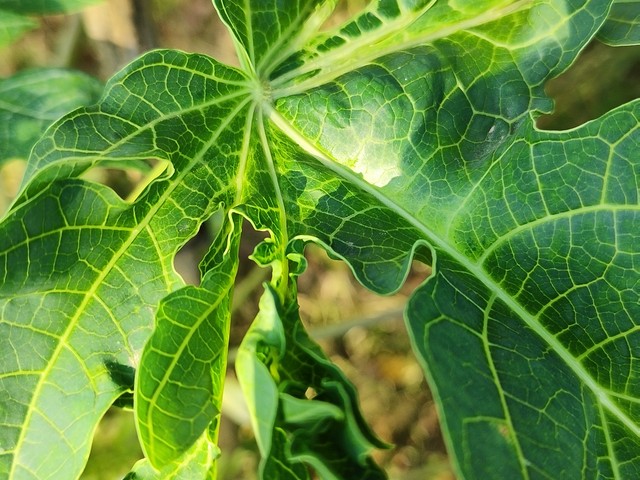

Supplement: Supplementary file 1 [file mmc1.zip › Demo dataset/Original Images/Curl/Curl(6).jpg]

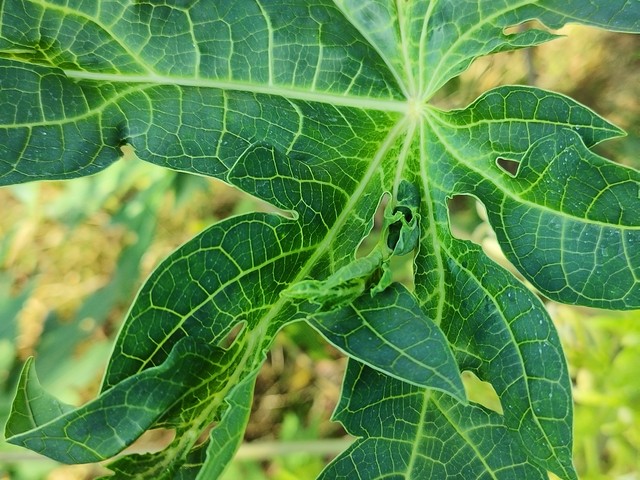

Supplement: Supplementary file 1 [file mmc1.zip › Demo dataset/Original Images/Curl/Curl(7).jpg]

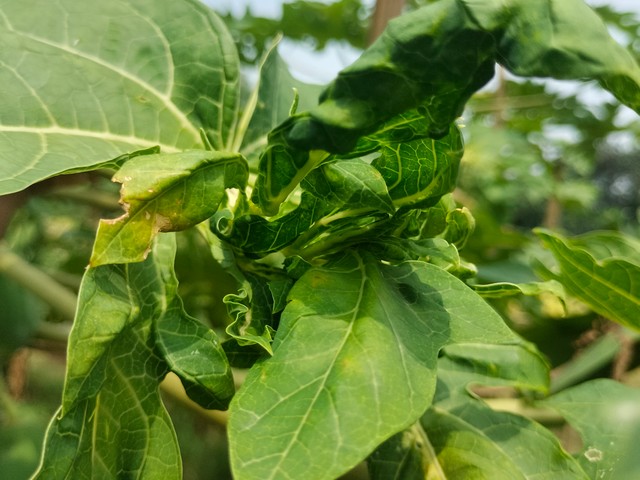

Supplement: Supplementary file 1 [file mmc1.zip › Demo dataset/Original Images/Curl/Curl(8).jpg]

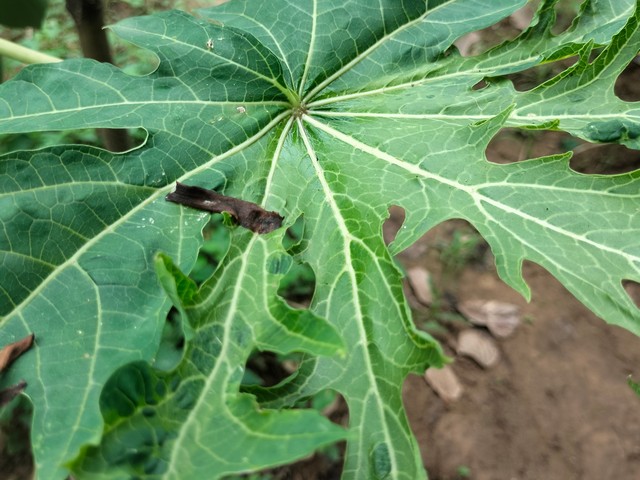

Supplement: Supplementary file 1 [file mmc1.zip › Demo dataset/Original Images/Curl/Curl(9).jpg]

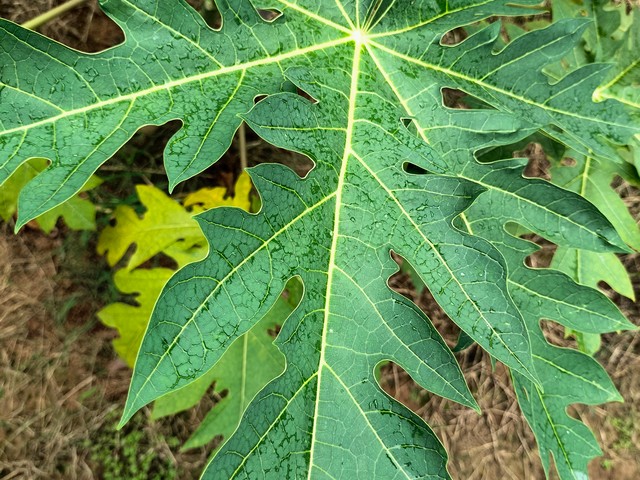

Supplement: Supplementary file 1 [file mmc1.zip › Demo dataset/Original Images/Healthy/Healthy(1).jpg]

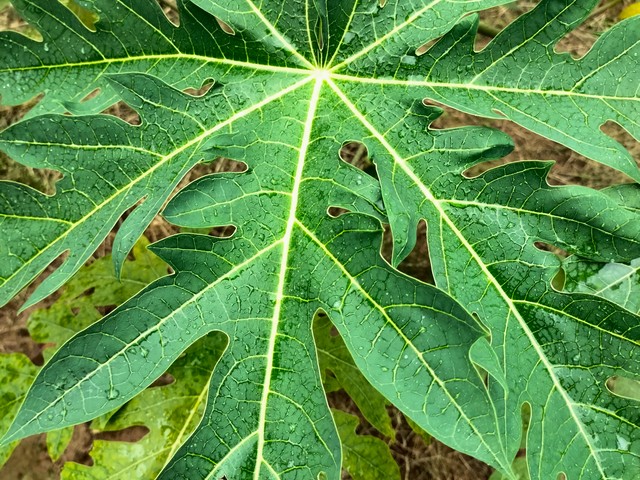

Supplement: Supplementary file 1 [file mmc1.zip › Demo dataset/Original Images/Healthy/Healthy(10).jpg]

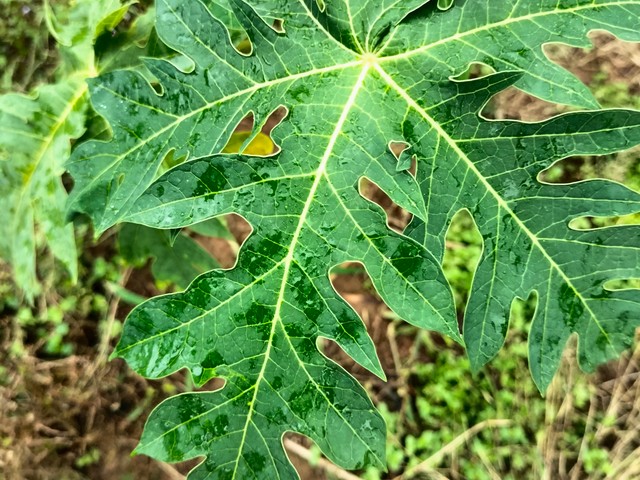

Supplement: Supplementary file 1 [file mmc1.zip › Demo dataset/Original Images/Healthy/Healthy(2).jpg]

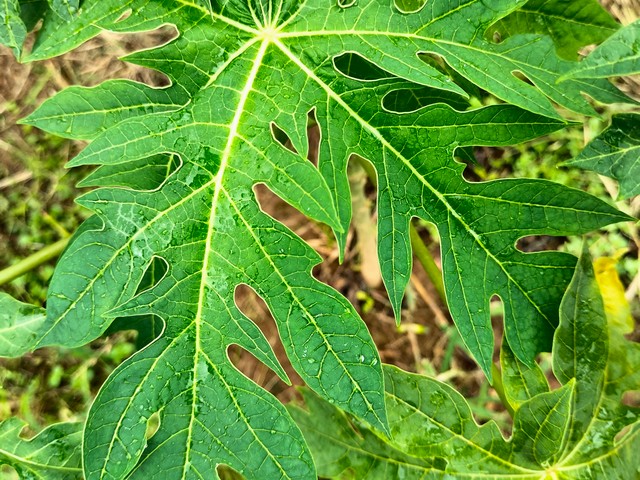

Supplement: Supplementary file 1 [file mmc1.zip › Demo dataset/Original Images/Healthy/Healthy(3).jpg]

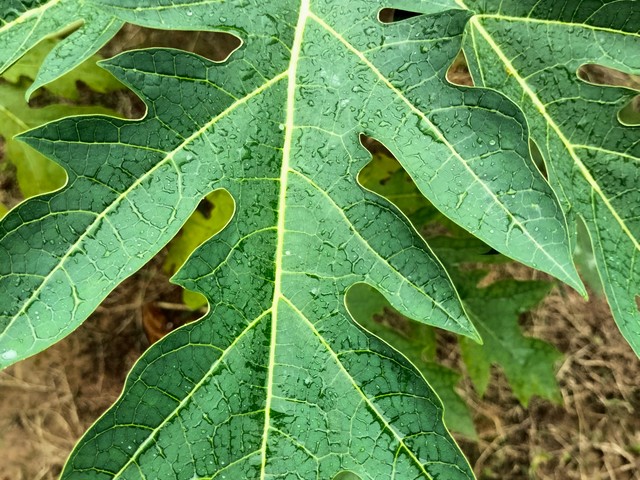

Supplement: Supplementary file 1 [file mmc1.zip › Demo dataset/Original Images/Healthy/Healthy(4).jpg]

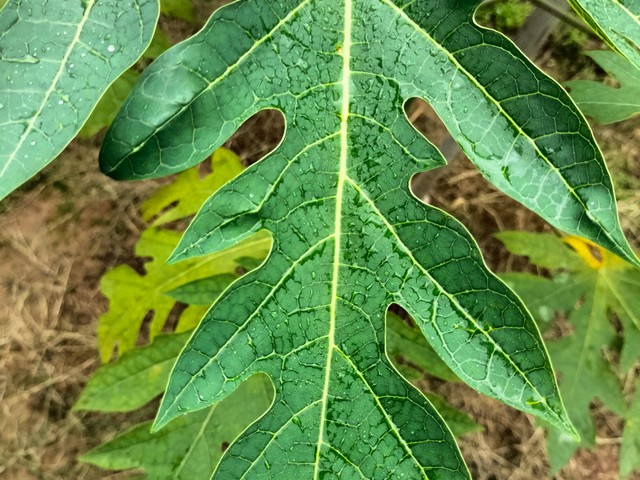

Supplement: Supplementary file 1 [file mmc1.zip › Demo dataset/Original Images/Healthy/Healthy(5).jpg]

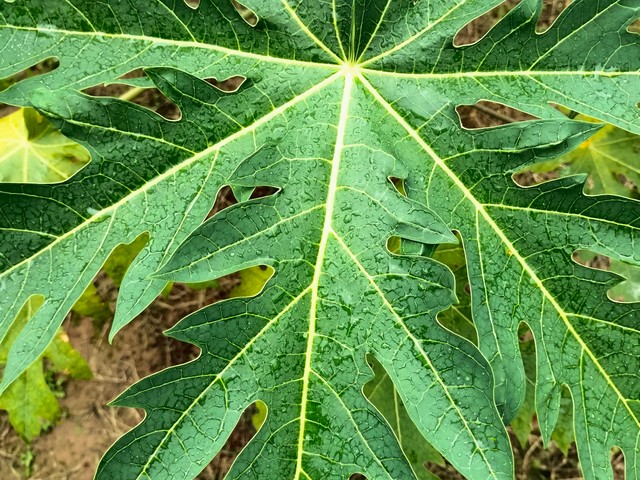

Supplement: Supplementary file 1 [file mmc1.zip › Demo dataset/Original Images/Healthy/Healthy(6).jpg]

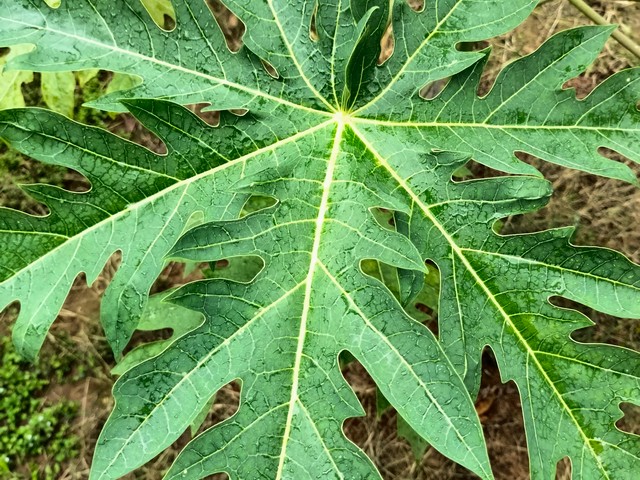

Supplement: Supplementary file 1 [file mmc1.zip › Demo dataset/Original Images/Healthy/Healthy(7).jpg]

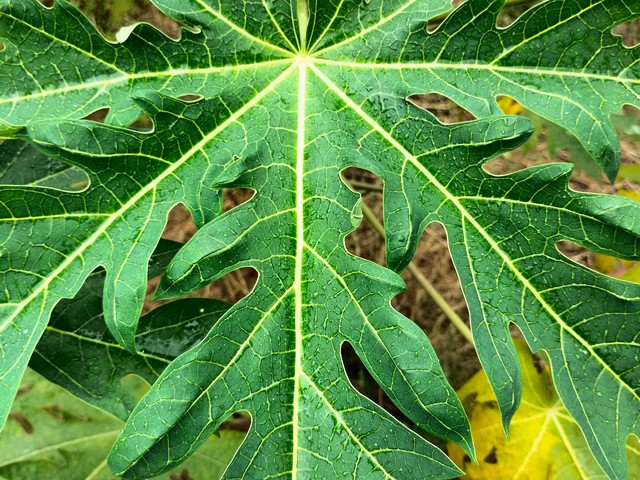

Supplement: Supplementary file 1 [file mmc1.zip › Demo dataset/Original Images/Healthy/Healthy(8).jpg]

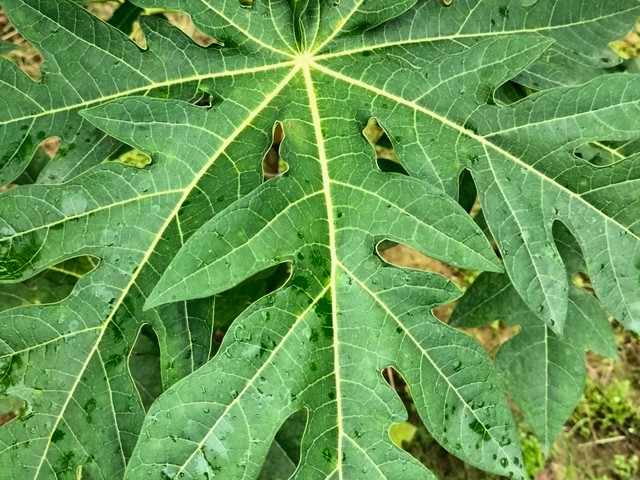

Supplement: Supplementary file 1 [file mmc1.zip › Demo dataset/Original Images/Healthy/Healthy(9).jpg]

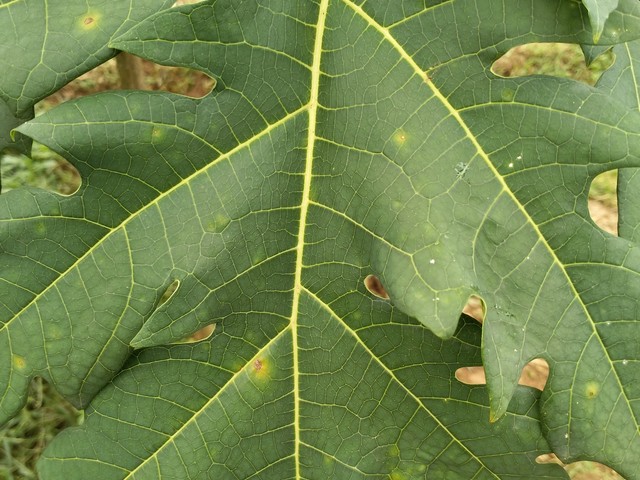

Supplement: Supplementary file 1 [file mmc1.zip › Demo dataset/Original Images/RightSpot/RingSpot(1).jpg]

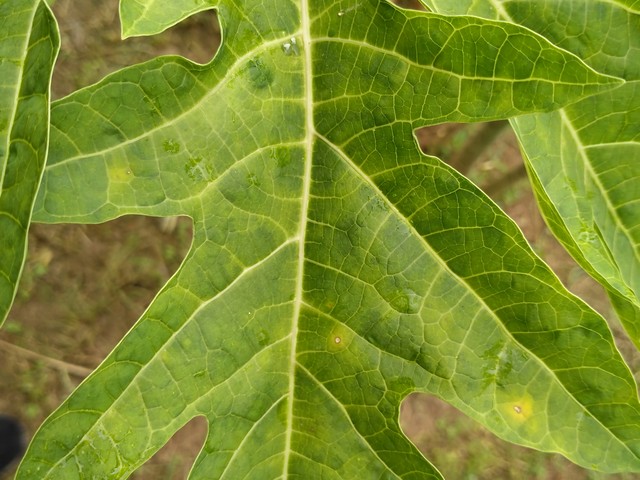

Supplement: Supplementary file 1 [file mmc1.zip › Demo dataset/Original Images/RightSpot/RingSpot(10).jpg]

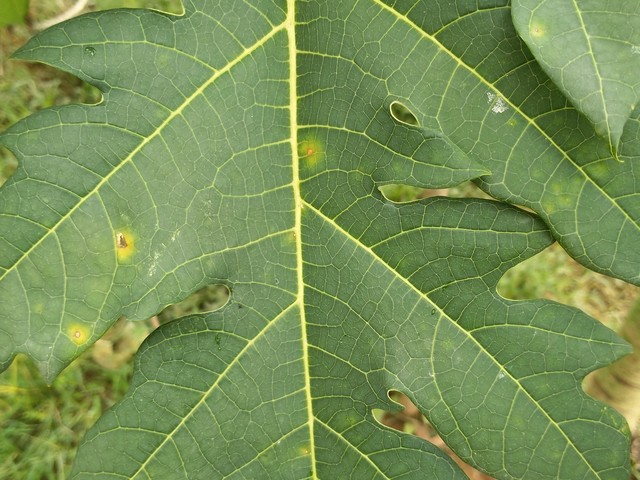

Supplement: Supplementary file 1 [file mmc1.zip › Demo dataset/Original Images/RightSpot/RingSpot(2).jpg]

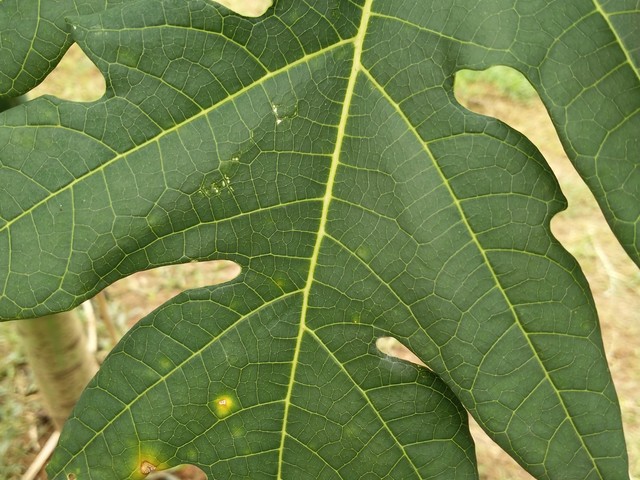

Supplement: Supplementary file 1 [file mmc1.zip › Demo dataset/Original Images/RightSpot/RingSpot(3).jpg]

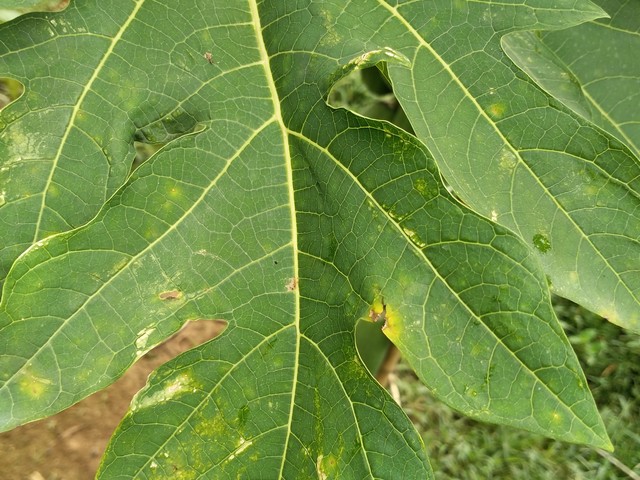

Supplement: Supplementary file 1 [file mmc1.zip › Demo dataset/Original Images/RightSpot/RingSpot(4).jpg]

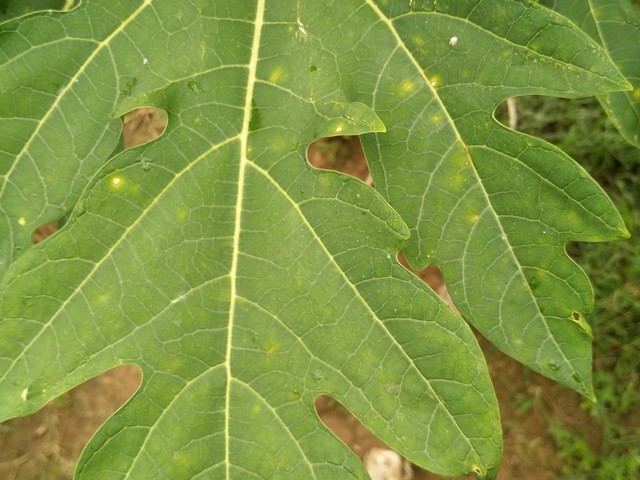

Supplement: Supplementary file 1 [file mmc1.zip › Demo dataset/Original Images/RightSpot/RingSpot(5).jpg]

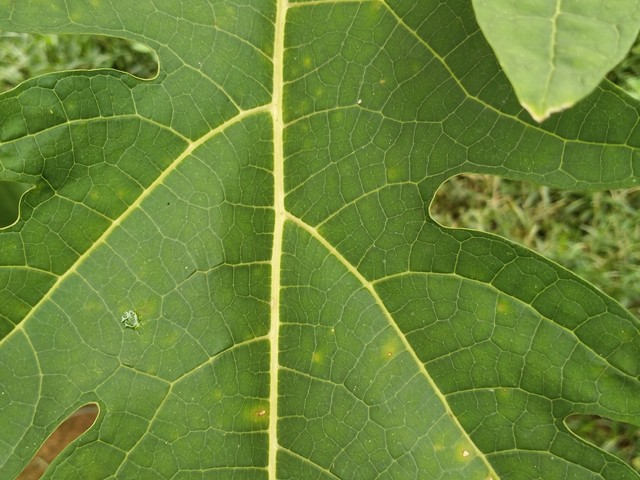

Supplement: Supplementary file 1 [file mmc1.zip › Demo dataset/Original Images/RightSpot/RingSpot(6).jpg]

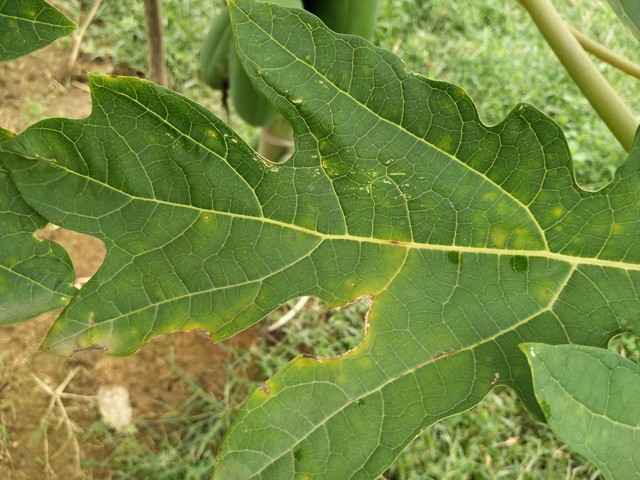

Supplement: Supplementary file 1 [file mmc1.zip › Demo dataset/Original Images/RightSpot/RingSpot(7).jpg]

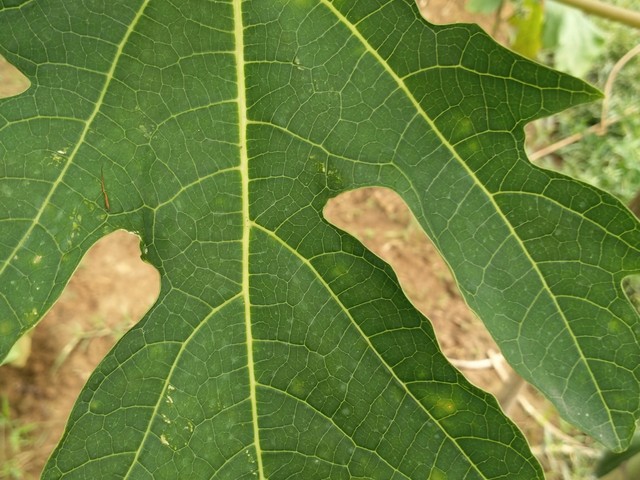

Supplement: Supplementary file 1 [file mmc1.zip › Demo dataset/Original Images/RightSpot/RingSpot(8).jpg]

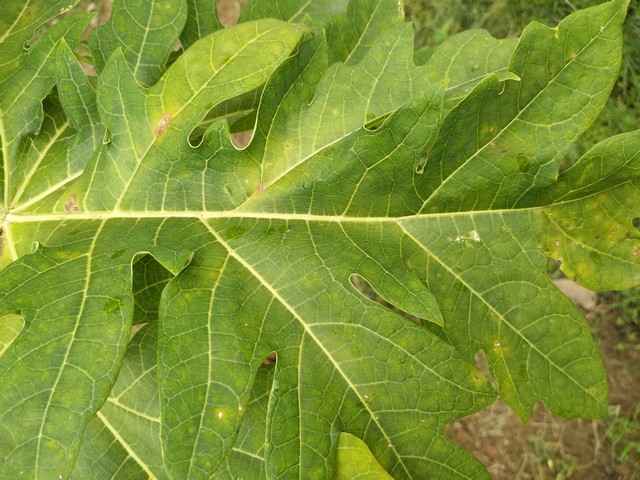

Supplement: Supplementary file 1 [file mmc1.zip › Demo dataset/Original Images/RightSpot/RingSpot(9).jpg]
